# Incidence of suicide among adolescent and young adult cancer patients: a population-based study

**DOI:** 10.1186/s12935-021-02225-y

**Published:** 2021-10-18

**Authors:** Pengcheng Yang, Lei Zhang, Xiaohua Hou

**Affiliations:** Division of Gastroenterology, Union Hospital, Tongji Medical College, Huazhong University of Science and Technology, Jiefang Avenue 1277, Wuhan, 430022 China

**Keywords:** Suicide, Cancer, Adolescent and young adult (AYA), Non-cancer cause of death, Surveillance Epidemiology and End Results (SEER)

## Abstract

**Background:**

As the survival rates of cancer patients continue to increase, most cancer patients now die of non-cancer causes. Several studies have been showing elevated suicide rates among patients with cancer. However, no large-scale study has thoroughly assessed the risk of suicide among adolescent and young adult (AYA) patients with cancer. This study was conducted to characterize suicide mortality among AYA patients in the US and identify risk factors associated with a higher risk of suicide.

**Methods:**

Patients aged 15–39 years were residents of the US served by the Surveillance, Epidemiology, and End Results (SEER) program, who were diagnosed with cancers from 1973 to 2015.

**Results:**

We report that 981 of the 572,500 AYA patients with cancer committed suicide, for an age-, sex-, and race-adjusted suicide rate of 17.68/100,000 person-years. The rate of suicide was 14.33/100,000 person-years in the corresponding general population, giving a standardized mortality ratio (SMR) of 1.234 [95% confidence interval (CI) 1.159–1.313]. Higher suicide rates were associated with male sex, white race, unmarried state, distant tumor stage, and single primary tumor. AYA patients with otorhinolaryngologic, gonad, stomach, soft tissue, and nasopharyngeal cancer were at the greatest risk of suicide compared with those with other types of cancer. In older patients (≥ 40 years), the risk was highest in those with lung, stomach, oral cavity and pharynx, larynx, and bone malignancies. SMRs were highest in the first 5 years after diagnosis for most types of cancer.

**Conclusion:**

AYA patients with cancer in the US have over 20% higher the incidence of suicide of the general population, and most suicides occurred in the first 5 years following diagnosis. Suicide rates vary among patients with cancers of different anatomic sites. Further examination of the psychological experience of these young patients with cancer, particularly that of those with certain types of cancer, is warranted.

**Supplementary Information:**

The online version contains supplementary material available at 10.1186/s12935-021-02225-y.

## Introduction

As the survival rates of cancer patients continue to increase, most patients with cancer now die of non-cancer causes [1]. Several studies have been showing elevated suicide rates among patients with cancer [2–6]. The American College of Surgeons Committee on Cancer, the American Society of Clinical Oncology, and the National Comprehensive Cancer Network have identified emotional distress and anxiety as vital signs of patients with cancer [7, 8]. Suicide is the culmination of unmanaged distress, which is the 10th leading cause of death in the US, and risk factors for suicide among patients with cancer are similar to those in the general population, including male sex and older age [9, 10]. The incidence of suicide in patients with cancer in the US is approximately twice that of the general population of the US [10]. It will become crucial to identify patients with cancer at elevated risk of suicide. However, these population-based studies have not distinguished between childhood, adolescent and young adults (AYA), and older adults, confounding the discrepancy of the incidence of suicide among these different age groups.

AYA, 15–39 years old, whose physical, psychosocial, and psychical conditions differ from other age groups, are at an early stage in life when their education, career, and family life just start, and they have a long way to go in their lives. It is devastating for AYA, physically and mentally, to be diagnosed with cancer. AYA cancer survivors face a unique set of challenges after diagnosis, including the ability to maintain their work and educational goals during and after the diagnosis and cancer treatment [11, 12]. Additionally, cancer incidence increased faster in AYAs than in the younger and older population [13]. Every year, approximately 70,000 AYAs are diagnosed with cancer in the United States and are the leading cause of disease-related death in the AYA population [14]. Nowadays, AYA patients with cancer lose the advantage of prognosis compared to older cancer patients, and the survival quality of AYA patients with cancer should be focused on [15]. Suicide among AYA patients with cancer, a special population in both demographic and pathology, warranted attention. The clinical detection of suicide is poor [16]; moreover, there is currently no contemporary research to assist clinicians in identifying AYA patients with cancer at high risk of suicide.

This study aimed to present a contemporary analysis of the risk of suicide among AYA patients with cancer through the National Cancer Institute Surveillance, Epidemiology, and End Results (SEER) repository freely available to the public. The results of this retrospective cohort study suggest that suicide-prevention strategies may be aimed at AYA patients with cancer of otorhinolaryngologic neoplasms (exclusive of nasopharyngeal cancer), gonad, stomach, and soft tissue; as well as nasopharyngeal cancer, and osseous and chondromatous tumor. The result of this study may be used clinically by oncologists and psychiatrists to create survivorship programs to reduce distress and anxiety and mitigate the risk of suicide among AYA patients with cancer.

## Methods

### Data resource

AYAs and older adults with primary cancer diagnosed between 1973 and 2015 were identified from the Surveillance, Epidemiology, and End Results (SEER-18 registry) program of the National Cancer Institute. SEER is a network of population-based incident tumor registries from geographically distinct regions in the US, covering 28% of the US population. The SEER registry includes data on age at diagnosis, sex, race, marital status, and year of diagnosis. Comparisons between patients with cancer and the general US AYA population were based on mortality data from 1969 to 2015, accessed through the SEER program. We extract the number of suicides and deaths from all causes from the SEER-9 registry to evaluate the trend of suicide in all causes of death from 1973 to 2015.

It should be noted that the database did not record patients' personal private information, such as name, address, and contact information. Therefore, the authors had no access to information that could identify individual participants during or after data collection. This study was approved by the Institutional Review Board of the Union Hospital of Huazhong University of Science and Technology.

### Inclusion criteria

We include all patients with a cancer diagnosis, except those whose information was obtained only from death certificate or autopsy. For anatomic site-specific analysis, AYAs with multiple primary tumors were excluded because suicides in these patients were not due to a single anatomic site of cancer. We adopted the AYA Site Recode/WHO 2008 definition for cancer classification, and analyses were presented for patients who accrued 10,000 person-years or more of survival time in the SEER registry. For older adults, Site Recode ICD-O-3 (International Classification of Disease for Oncology, the third edition) was chosen for classification. The leukemias were analyzed as an aggregate group.

### Variables

The study variables included demographic and clinical characteristics. Demographic variables such as age at diagnosis, race, sex, year of diagnosis, and marital status were selected. Clinical characteristics included stage of cancer (in situ, localized, regional, and distant), anatomic site of the disease, cause of death, and vital status at last follow-up; SEER does not code comorbidities or diagnoses associated with suicide, including suicidal ideation, previous suicide attempts, or use of antidepressive medications; these data were unavailable.

We considered AYA patients to have committed suicide if the cause of death variable was recorded as “Suicide and Self-inflicted Injury.” Patients with other causes of death, including “Accidents and Adverse Effects,” “Homicide and Legal Intervention,” and “Other Cause of Death,” were excluded.

The survival time was counted in months, and the smallest value was 1 month. The SEER data set coded patients whose survival time was < 1 month as surviving for zero. Based on the standard epidemiological convention[17], we assigned these patients a survival time of 0.5 month to analysis.

### Statistical analyses

We calculated suicide rates (number of suicides divided by person-years of survival) standardized to age, sex, and race distribution, as well as to compare rates by different anatomic sites. Age at diagnosis was partitioned into 5-year intervals for standardization. We adjusted analyses that address the relation of suicide and demographic characteristics to the age distribution at diagnosis. Standardized mortality ratios (SMR, suicide rate of patients divided by suicide rate of the comparison population) and 95% confidence intervals (CIs) were calculated as described in previous studies [17–19], adjusted for age, sex, and race to the general US population. All statistical analyses were performed using statistical software SEER*Stat 8.3.5, STATA 15.0, and R × 64 3.5.2.

## Results

**Fig. 1:**
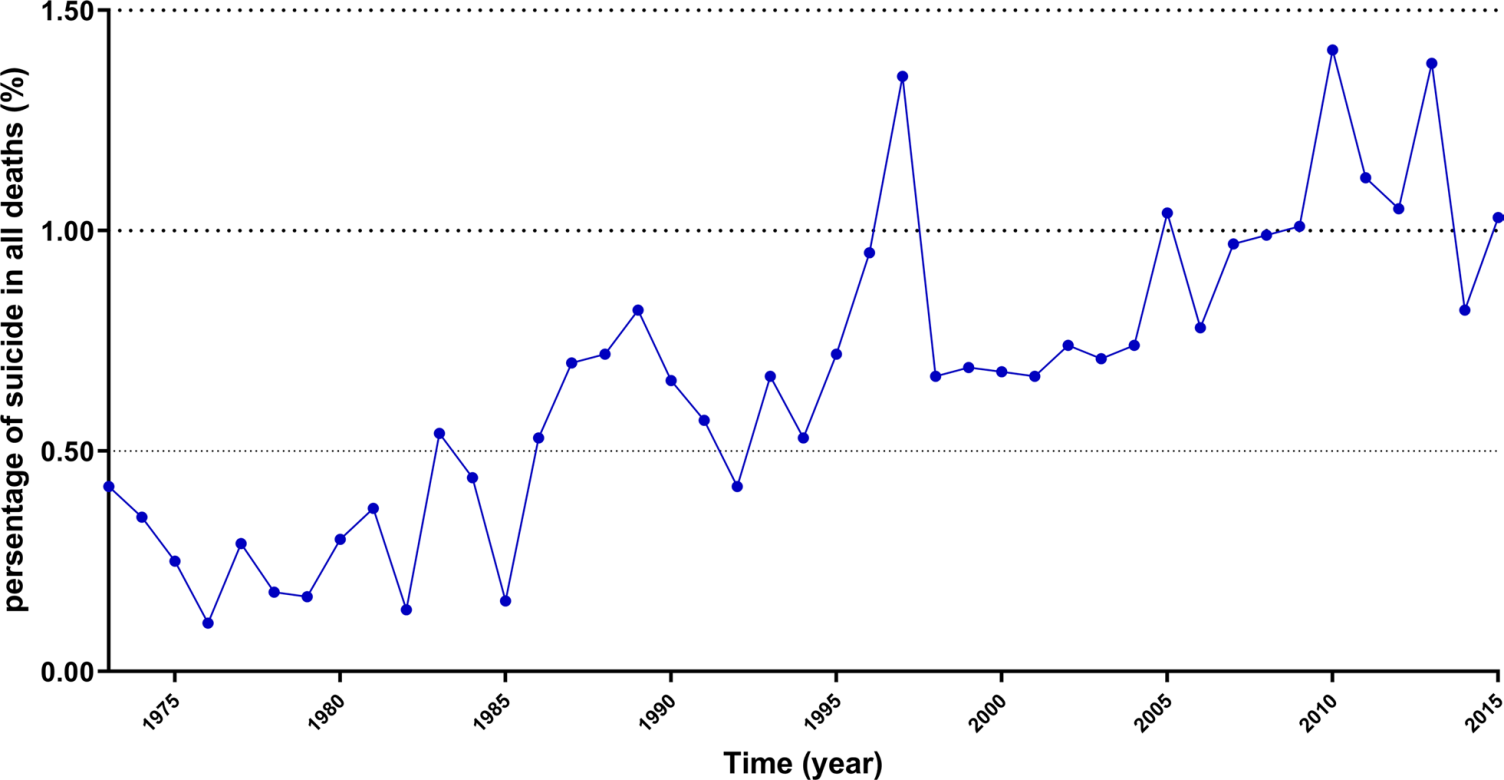
Trends in suicide incidence over time. Despite the fluctuations, there was a trend of increase in the percentage of AYA patients who committed suicide in all deaths since the 1970s through 2015

In total, 981 suicides were identified in 572,500 AYA patients with cancer. The suicide rate was 17.68/100,000 person-years after adjusting for age, sex, and race. Consequently, the suicide rate in the general US AYA population was 14.33 per 100,000 person-years. It gave an SMR of 1.234 (95%CI, 1.159–1.313). We calculated the survival time of all AYA patients in the SEER (range, 0–42.92 years), with a mean survival time of 9.69 years, regardless of the cause of death. There was a trend of an increase in the percentage of AYA patients who committed suicide in all deaths since the 1970s through 2015 (Fig. 1).

### Characteristics relate to higher suicide rates

The predominant patients who committed suicide were male, white, and unmarried. Higher suicide rates were associated with advanced tumors at diagnosis and single primary tumor (Table 1). Higher rates of suicide were noted with increasing age at diagnosis among males. Among all AYA patients, the age of 20–24 years at diagnosis have the highest rates of suicide, and the youngest patients (15–19 years) had the lowest rates of suicide (Additional file 2: Table S1)

**Table 1:** Incidence of suicide among AYA cancer patients by demographic and tumor characteristics

Characteristic	Patients with cancer		Suicide		Suicide per 100,000 Person-years ^a^	SMR ^ab^	95% CI
	No	%	No	%			
Sex							
Male	223,123	38.97%	658	67.07%	33.75	1.484	1.374–1.601
Female	349,377	61.03%	323	32.93%	9.01	1.449	1.299–1.616
Race							
White	455,982	79.65%	901	91.85%	19.55	1.27	1.189–1.355
Black	58,305	10.18%	34	3.47%	7.46	0.78	0.558–1.092
Other	58,213	10.17%	46	4.69%	9.83	1.039	0.778–1.387
Material status							
Married	259,679	45.36%	376	38.33%	13.13	0.908	0.821–1.005
Unmarried	263,589	46.04%	519	52.91%	24.09	1.644	1.508–1.792
Unkmon	49,232	8.60%	86	8.77%	18.72	1.312	1.062–1.621
Stage at presentation							
In situ	43,246	7.55%	66	6.73%	13.48	0.927	0.728–1.180
Localized	214,419	37.45%	386	39.35%	14.76	1.019	0.922–1.126
Regional	99,829	17.44%	142	14.48%	15.23	1.06	0.899–1.249
Distant	65,836	11.50%	66	6.73%	20.87	1.444	1.134–1.838
Unstaged/Unknown	149,170	26.06%	321	32.72%	29.28	1.94	1.739–2.164
No of primary							
Single	475,758	83.10%	852	86.85%	19.07	1.332	1.246–1.425
Multiple	96,742	16.90%	129	13.15%	11.67	0.828	0.697–0.984
Year of diagnosis							
1973–1983	50,407	8.80%	182	18.55%	16.64	1.176	1.017–1.360
1984–1994	89,064	15.56%	322	32.82%	22.28	1.552	1.391–1.731
1995–2005	184,245	32.18%	314	32.01%	15.63	1.085	0.971–1.211
2006–2015	248,784	43.46%	163	16.62%	16.27	1.136	0.975–1.325
All AYA patients with cancer	572,500	1	981	1	17.68	1.234	1.159–1.313

*SEER* Surveillance, epidemiology, and end results, *SMR* standardized mortality ratio

^a^Adjusted to the age distribution in the population served by the SEER program.

^b^For the categories of sex and race, SMR reference population was the specific category in the general US subpopulation (eg, the SMR for male AYAs is the observed number of suicides in male AYA patients divided by the expected number of suicides based on the rate in men in the general AYA population). For marital status, stage at presentation, number of primary tumors, and year of diagnosis, SMR reference population is the entire general US AYA population from 1969 through 2015.

### Tumor sites associated with higher suicide rates

A total of 852 suicides were observed among 475,758 AYAs with a single primary tumor, accumulating 4,473,748.2 person-years. AYA patients with most types of cancer (thyroid cancer was an exception) have higher suicide rates than the general US population. The highest suicide rates were observed among AYA patients with cancer of otorhinolaryngologic neoplasms (exclusive of nasopharyngeal cancer) (SMR, 4.002; 95%CI, 2.083–7.692), followed by cancer of gonad (SMR, 1.939; 95% CI, 1.009–3.726), stomach cancer (SMR, 2.965; 95%CI, 1.234–7.124), cancers of soft tissue (SMR, 3.058; 95%CI, 2.479–3.773), and nasopharyngeal cancer (SMR, 3.139; 95%CI, 1.410–6.987). There were sex differences in the anatomic sites associated with higher suicide rates. Male AYA patients with cancers of the nasopharyngeal, gonad, soft tissue, stomach, and otorhinolaryngologic neoplasms (excluding nasopharyngeal cancer) had higher suicide rates. Among females, patients with cancer of otorhinolaryngologic neoplasms (excluding nasopharyngeal cancer), osseous and chondromatous, stomach, and nervous system were associated with higher suicide risk (Table 2).

**Table 2:** Suicide rates by anatomic site of cancer

Site^a^	No of suicides	No of patients	Survival time (person-yeats)	Overall(male and female)			Male AYA patients^e^			Female AYA patients^e^		
				Suicide rate^bc^	SMR^cd^	CI^cd^	Suicide rate^bc^	SMR^cd^	CI^cd^	Suicide rate^bc^	SMR^cd^	CI^cd^
Lung and bronchus	9	8029	34,610.2	22.79	1.90	0.986–3.651	35.28	1.69	0.759–3.762	15.34	2.53	0.815–7.835
Stomach	5	4294	12,559.5	37.12	2.97	1.234–7.124	65.01	2.28	0.736–7.079	20.5	5.38	1.344–21.495
Oral cavity and pharynx	25	7020	74,198.6	29.23	2.28	1.538–3.369	51.39	2.25	1.452–3.489	16.02	2.39	0.993–5.730
Hodgkin lymphoma	66	25,924	300,315.1	17.33	1.56	1.223–1.981	34.17	1.58	1.216–2.063	7.29	1.43	0.794–2.587
Kidney	9	8327	62,117.5	23.76	0.93	0.483–1.784	49.21	0.88	0.418–1.840	8.59	1.17	0.292–4.663
Thyroid	45	52,147	548,098.5	11.04	0.89	0.666–1.194	21.14	1.00	0.662–1.499	5.02	0.80	0.529–1.220
Nervous system	42	19,736	142,982.9	30	2.04	1.510–2.764	46.93	1.90	1.345–2.690	19.91	2.68	1.440–4.975
Non-hodgkin lymphoma	53	27,002	206,747.1	19.6	1.66	1.267–2.171	38.21	1.71	1.280–2.282	8.51	1.39	0.663–2.916
Leukemias	19	20,857	117,841.5	15.76	1.11	0.707–1.739	21.17	0.83	0.472–1.465	12.54	2.59	1.235–5.432
Colorectal	40	20,568	143,728.9	28.26	1.95	1.427–2.652	44.23	1.87	1.309–2.677	18.73	2.21	1.187–4.101
Bladder	17	4433	64,280.5	17.56	1.41	0.875–2.263	28.31	1.37	0.823–2.266	11.15	1.81	0.454–7.254
Nasopharyngeal carcinoma	6	1747	14,923.8	33.51	3.14	1.410–6.987	89.73	3.63	1.628–8.069	0	0.00	—
Gonad	9	6501	68,776.8	39	1.94	1.009–3.726	87.33	5.04	1.261–20.167	10.2	1.65	0.786–3.459
Breast	53	58,699	547,036.5							8.81	1.45	1.104–1.891
Cervix and uterus	43	29,976	341,688.2							12.02	1.95	1.447–2.630
Naso cav,mid ear,sinus,larynx	9	1417	13,479	55.91	4.00	2.083–7.692	54.62	3.11	1.395–6.912	56.68	9.49	3.060–29.410
Osseous and chondrommatous	17	6359	53,608	33.16	2.33	1.446–3.743	41.32	1.95	1.109–3.440	28.3	4.30	1.788–10.322
Soft tissue	87	26,070	188,201.8	34.2	3.06	2.479–3.773	72.81	3.24	2.595–4.044	11.18	2.06	1.072–3.959
Germ cell and trophoblastic	128	39,712	454,603	15.08	1.32	1.109–1.568	28.74	1.31	1.097–1.560	6.93	1.78	0.668–4.741
Melanoma and skin carcinomas	91	45,699	539,030.5	15.03	1.28	1.040–1.569	27.39	1.27	0.989–1.621	7.67	1.30	0.900–1.888
All AYA patients with single primary tumor	852	475,758	4,473,748.2	19.04	1.55	1.447–1.655	34.47	1.54	1.417–1.669	9.85	1.57	1.393–1.763
General US AYA population				12.31	1.00	Reference	22.42	1.00	Reference	6.28	1.00	Reference

*SMR* standardized mortality ratio

^a^Analysis was limited to tumor sites for which at least 100,000 person years were accrued

^b^Per 100,000 person-years

^c^Adjusted to the age, race, and sex distributions of patients with a single primary tumor

^d^Reference population: general US AYA population, 1969 to 2015

^e^Sex-specific analysis, adjusted for age and race distributions of patients with single primary tumor

### Suicide risk with time after diagnosis

The relative risk of suicide (SMR) among AYA patients with cancer was highest in the first 5 years after diagnosis and then gradually decreased. Over fifteen years after diagnosis, the suicide risk did not show any apparent differences between AYA patients and the general US population. The relative suicide risks subside with time in AYA patients with kidney, non-Hodgkin lymphoma, gonad, breast, cervix and uterus, soft tissue, and nasopharyngeal tumors. In contrast, for certain cancers, the SMR of suicide remained elevated over time or increased after a decrease (e.g., cancer of the lip, oral cavity, and pharynx, Hodgkin lymphoma, leukemias, osseous, and chondromatous) (Table 3).

**Table 3:** Suicide in Patients With Cancer by Site and Years Since Diagnosis

Cancer site^a^	0–5 years	5–10 years	10–15 years	15–30 years
All				
No of suicide	352	194	132	153
Person-years	2,413,420.2	1,785,358.3	1,369,803.9	1,264,207.9
SMR (95% CI)^bc^	1.199 (1.080–1.331)	0.903 (0.785–1.040)	0.804 (0.678–0.954)	1.012(0.864–1.185)
Lung and bronchus				
No of suicide	4	1	3	1
Person-years	21,310.5	12,794.1	10,188	9643.1
SMR (95% CI)^bc^	1.348 (0.506–3.593)	0.572 (0.081–4.062)	2.168 (0.699–6.772)	0.763 (0.107–5.413)
Stomach				
No of suicide	5	0	0	0
Person-years	7945.9	/	/	/
SMR (95% CI)^bc^	4.223 (1.758–10.146)	/	/	/
Oral cavity and pharynx				
No of suicide	9	6	4	6
Person-years	40,367.3	31,639.7	25,408.4	24,024.3
SMR (95% CI)^bc^	1.496 (0.779–2.876)	1.279 (0.575–2.846)	1.06 (0.398–2.823)	1.683 (0.756–3.747)
Larynx				
No of suicide	4	1	0	4
Person-years	7397.3	5524.3	/	4105.4
SMR (95% CI)^bc^	3.257 (1.223–8.679)	1.103 (0.155–7.833)	/	5.895 (2.213–15.708)
Hodgkin lymphoma				
No of suicide	19	15	14	15
Person-years	154,529.1	121,706.9	95,506.2	90,800.8
SMR (95% CI)^bc^	0.882 (0.562–1.383)	0.884 (0.533–1.467)	1.048 (0.620–1.769)	1.178 (0.710–1.954)
Kidney				
No of suicide	5	3	1	0
Person-years	34,213.4	21,394.6	13,296.1	/
SMR (95% CI)^bc^	0.916 (0.381–2.202)	0.881 (0.284–2.732)	0.476 (0.067–3.376)	/
Thyroid				
No of suicide	10	15	5	11
Person-years	304,940.9	231,914.4	177,708.3	163,000.3
SMR (95% CI)^bc^	0.36 (0.194–0.669)	0.705 (0.425–1.170)	0.305 (0.127–0.732)	0.725 (0.401–1.309)
Nervous system				
No of suicide	18	11	7	6
Person-years	78,879.9	48,173.6	32,588	28,791.4
SMR (95% CI)^bc^	1.598 (1.007–2.537)	1.671 (0.926–3.018)	1.604 (0.765–3.365)	1.569 (0.705–3.493)
Non-hodgkin lymphoma				
No of suicide	30	10	9	4
Person-years	107,191.8	74,337.6	52,627.9	44,459
SMR (95% CI)^bc^	1.789 (1.251–2.559)	0.868 (0.467–1.613)	1.102 (0.573–2.118)	0.583 (0.219–1.552)
Leukemias				
No of suicide	12	3	1	3
Person-years	66,781.5	37,020.2	23,586.8	18,850.3
SMR (95% CI)^bc^	1.242 (0.705–2.187)	0.569 (0.183–1.763)	0.3 (0.042–2.130)	1.134 (0.366–3.516)
Colorectal				
No of suicide	24	6	6	4
Person-years	83,362.7	52,617.1	38,488.4	34,003.3
SMR (95% CI)^bc^	1.981 (1.328–2.956)	0.799 (0.359–1.778)	1.111 (0.499–2.473)	0.853 (0.320–2.273)
Bladder				
No of suicide	5	5	2	3
Person-years	36,080.1	31,857.8	28,210.3	28,337.9
SMR (95% CI)^bc^	0.754 (0.314–1.813)	0.854 (0.355–2.051)	0.386 (0.096–1.542)	0.574 (0.185–1.779)
Gonad				
No of suicide	4	2	2	1
Person-years	41,069.2	32,830.5	28,353.8	28,643.1
SMR (95% CI)^bc^	1.498 (0.562–3.992)	0.947 (0.237–3.786)	1.108 (0.277–4.430)	0.551 (0.078–3.909)
Breast^d^				
No of suicide	21	16	10	6
Person-years	302,555.6	209,432.5	154,023.4	140,039
SMR (95% CI)^bc^	1.023 (0.667–1.569)	1.124 (0.689–1.835)	0.956 (0.515–1.778)	0.632 (0.284–1.406)
Cervix and uterus^d^				
No of suicide	19	9	7	6
Person-years	190,234.6	154,903.8	129,910.9	126,362.5
SMR (95% CI)^bc^	1.556 (0.992–2.439)	0.91 (0.474–1.749)	0.848 (0.404–1.780)	0.75 (0.337–1.668)
Nasopharyngeal				
No of suicide	5	0	0	1
Person-years	8107.6	/	/	3563.3
SMR (95% CI)^bc^	3.836 (1.597–9.217)	/	/	1.832 (0.258–13.009)
Osseous and chondrommatous				
No of suicide	8	2	3	3
Person-years	30,477	21,211.8	16,519.3	15,391.4
SMR (95% CI)^bc^	1.948 (0.974–3.895)	0.708 (0.177–2.831)	1.368 (0.441–4.240)	1.458 (0.470–4.521)
Soft tissue				
No of suicide	53	14	10	9
Person-years	106,086.5	73,337.1	58,510.3	54,880.6
SMR (95% CI)^bc^	3.229 (2.467–4.227)	1.316 (0.779–2.222)	1.202 (0.647–2.235)	1.17 (0.609–2.248)
Germ cell and trophoblastic				
No of suicide	40	34	19	27
Person-years	236,632.7	189,951.7	152,340.3	145,582.2
SMR (95% CI)^bc^	0.821 (0.602–1.119)	0.872 (0.623–1.220)	0.61 (0.389–0.957)	0.909 (0.623–1.325)
Melanoma and skin carcinomas				
No of suicide	26	17	23	25
Person-years	284,288	231,236.7	184,811.7	174,225.1
SMR (95% CI)^bc^	0.726 (0.494–1.066)	0.587 (0.365–0.944)	0.991 (0.658–1.491)	1.139 (0.770–1.686)

*SMR* standardized mortality ratio

^a^Analysis was limited to tumor sites for which at least 10,000 person years were accrued

^b^Adjusted to the age and sex distributions of patients with single primary tumor

^c^Reference population: general US population, 1969 to 2015

^d^Sex-specific analysis, adjusted to the age distribution of patients with single primary tumor

### AYA cancer patients vs older adult patients

**Fig. 2:**
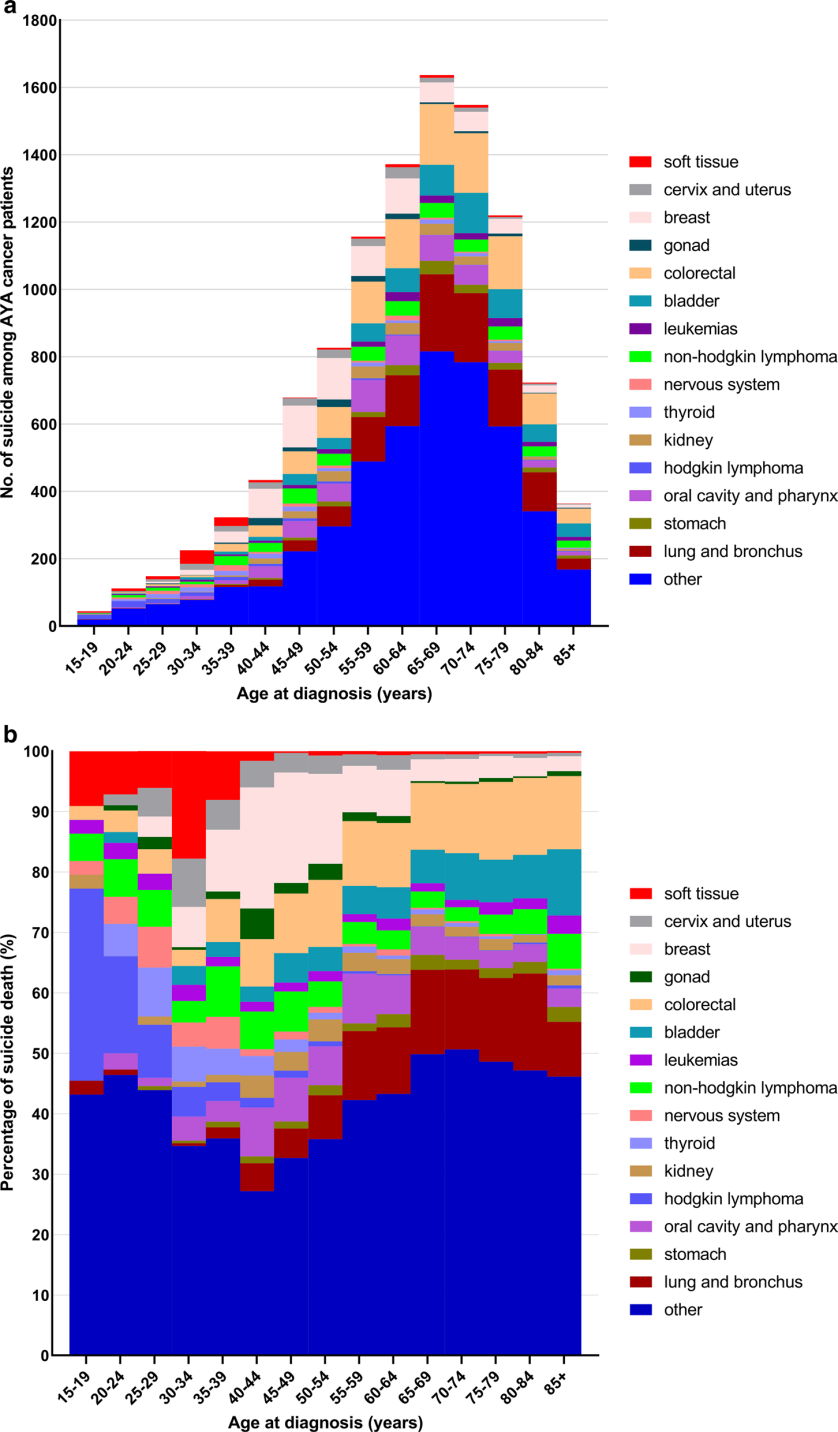
Distribution of suicides in cancer type among cancer patients as a function of age group. a The y-axis depicts the relative number of suicides compared to all cancer patients, and the x-axis depicts the age group at time of diagnosis. The colors depict the disease sites. For AYA patients, the plurality of suicides is seen in soft tissue cancer, and lymphoma. In contrast, among older adults (≥ 40 years old), the plurality of suicides occurs in patients with cancer of breast, lung and brounchs, and colorectum. b The y-axis depicts the absolute number of suicides and the x-axis depicts the age group at time of diagnosis. The colors depict the disease sites. The majority of suicide events are in patients diagnosed at an older age (≥ 40 years old), because most cancer patients are middle-aged and elderly

When comparing the two age groups, there were significant discrepancies in the distribution of suicide in sex, marital status, tumor stage, single or multiple, and year of diagnosis (Additional file 2: Table S2). For patients diagnosed at age < 40, the majority of suicide occurred in patients with soft tissue cancer and lymphoma. In contrast, among patients diagnosed at age ≥ 40, the majority of suicide occurred in patients with breast, lung and bronchus, and colorectal cancer (Fig. 2). The characteristics associated with a high suicide rate in the older adult patients were older age, male sex, white race, unmarried, distant tumor stage, and single primary cancer (Additional file 3: Table S2). The relative risk of suicide (SMR) in older adult patients vs. the general population was highest in those with cancer of lung and bronchus, stomach, oral cavity and pharynx, and larynx (Additional file 4: Table S3). Anatomical sites associated with a high relative risk of suicide differed between the two age groups (Additional file 1: Figure S1). Among patients diagnosed at ≥ 40 years of age, suicide rates were highest in patients with cancers of the lung and bronchus (SMR, 4.75; 95%CI, 4.49–5.04), followed by oral cavity and pharynx cancers (SMR, 3.52; 95%CI, 3.23–3.84), cancers of the stomach (SMR, 3.70; 95%CI, 3.20–4.29), cancers of the larynx (SMR, 3.05; 95%CI, 2.66–3.48), and nervous system tumors (SMR, 2.11; 95%CI, 1.63–2.75) (Additional file 4: Table S3). Generally, suicide rates and SMR were higher among older adult cancer patients compared with AYA patients. Contrary to older adult patients, the SMRs of female AYA patients were higher than those of males at most anatomic sites (Table 2, Additional file 4: Table S3).

## Discussion

We presented the first comprehensive analysis of the risk of suicide among AYA patients with cancer and found that the risk of suicide varied as a function of age, race, sex, marital status, disease site, and time after diagnosis. The incidence of suicide in AYA patients with cancer in the US was approximately 23.4% higher than that of the general US population. Similar to our study, previous studies revealed that patients with cancer had a higher risk of suicide, although the objects were not limited exclusively to adolescents and young adults [4, 10].

We observed that otorhinolaryngologic neoplasms (exclusive of nasopharyngeal cancer), gonad, stomach, soft tissue, and nasopharyngeal cancer were associated with the highest suicide rates among AYA patients. These findings differed from previous studies, and the sites associated with the highest risk vary between reports [3–5, 20–24]. Cancers of the head and neck had a significantly subversive impact on quality of life by affecting appearance and essential functions, such as speech, swallowing, and breathing [25]. Patients with head and neck cancer also had a high prevalence of depression [26]. A previous study reported that cancer had a negative impact on their ‘‘plans for having children,” with the possibility of infertility due to treatments such as chemotherapy, radiation, and certain types of surgery [27]. At the same time, the previous study also found a prominent impact of cancer diagnosed on sexual function in the younger group compared to the middle age group, highlighting the importance of this topic in survivorship research, particularly among AYA patients [27]. Our results are consistent with the study by Kjaer et al. [28] who reported an increased risk of suicide among Danish female patients with fertility problems. This may facilitate the comprehension of the high suicide rates of the AYA survivors with gonad tumors. The examination of psychological conditions in newly diagnosed patients with gastric cancer showed high levels of psychological distress [29]. Survivors of AYA soft tissue cancer suffered significant physical torture due to second malignant neoplasms and non-cancer causes, such as infectious and cardiovascular diseases [30]. Whether different age groups of patients with nasopharyngeal cancer had different survival outcomes remained controversial [31], another study found that younger patients (aged < 45) were more likely to have a higher risk of distant metastasis and poorer survival outcomes than older patients [32], and younger patients were more likely to have advanced disease at diagnosis [31].

Most cancer patients now die from non-cancer causes [1]. The results of this study suggested that suicide-prevention strategies may be aimed at those < 40 years of age with cancer of otorhinolaryngologic neoplasms (exclusive of nasopharyngeal cancer), gonad, stomach, soft tissue, and nasopharyngeal cancer. We found that characteristics associated with high suicide rates in the AYA cancer population, such as male sex, white race, and being unmarried, were similar to previous studies, which focused on cancer patients of all age groups. In contrast to previous studies [3, 5, 10], the sex disparity of SMR was not remarkable, and even the SMR of female AYA patients was slightly higher than that of the males. These studies concluded that males had appreciably higher relative suicide risk than females, but not observed in our research. Perhaps the relative risk of suicide had no apparent sex difference among AYA patients with cancer. The SMRs (suicide rate of patients divided by a suicide rate of the comparison population) of a certain population may not be compared to each other (only to the reference population), and these differences among studies may be due to different rates of suicide in the diverse reference populations.

Some studies reported that the relative risk of suicide among patients with cancer of all ages as an aggregated group was highest in the years immediately after diagnosis and decreased over time, but remained elevated for more than 15 years compared with the general population [10]. We could not conclude this from our data, which may be limited by the small number of suicides at most cancer sites.

We found that the suicide risk among AYA patients was lower than older adult patients with cancer. The types of cancer and their impact varied with age, and suicide characters among cancer survivors were different between AYA and older adult patients. AYA has better physical quality, fewer comorbidities, and a better response to treatment compared with older patients; therefore, AYA patients have a more promising prognosis. Unlike older age groups, AYAs are at an early stage of life when their education, career, and family life just get started and they have a long way to go in their lives. It is no doubt that being diagnosed with cancer is devastating for AYAs physically and mentally. Cancer treatment and quitting school/work after diagnosis were associated with AYA’s belief that cancer had a negative impact on school/work [33]. However, being diagnosed with cancer had a positive impact on their confidence in taking care of their health because AYAs with cancer likely would be managing and monitoring their health for many years [27]. Reasonably, the relative risks of suicide (SMR) of AYA cancer patients were lower than older adult patients.

There are some limitations to this study. First, the cause of death may be improperly recorded, especially suicide, which is often confused with homicide and accident events. Thus, we are unable to characterize the misclassification of suicide in the current work. Second, SEER does not code comorbidities or diagnoses associated with suicide, including suicidal ideation, previous suicide attempts, underlying diseases(like psychology disorder or use of antidepressive medications), and unhealthy living habits (tobacco and alcohol use). Distress, tobacco [34, 35] and alcohol use [36, 37] have been believed to be associated with an increased risk of suicide. The observed associations between cancer and suicide may be confounded by these factors, but we are unable to control for them. Finally, patients diagnosed in recent years have a short follow-up and a lower chance of death from any cause, including suicide.

## Conclusions

The results of this study suggest that suicide-prevention strategies may be aimed at those < 40 years of age with cancer of otorhinolaryngologic neoplasms (exclusive of nasopharyngeal cancer), gonad, stomach, soft tissue, and nasopharyngeal cancer. Psychological conditions among AYA cancer survivors deserve further investigation, particularly because the appropriate use of psychosocial interventions in patients with cancer can have a positive impact on quality of life. The role of lifestyle and comorbidity in determining risk of suicide among patients with cancer warranted further investigation. Finding the relationship between these factors and suicide may help develop survivorship in the AYA cancer population.

## Supplementary Information

**Additional file 1: Figure 1**. Comparison of suicide risk between AYA and older patients by cancer site. The distribution of suicide risk by tumor type was different between AYA patients and older patients. For AYA patients, SMRs of suicide were higher in those with cancers of colorectal, gonad, breast, cervix and uterus, and soft tissue. For older patients, higher SMRs of suicide were observed in those with cancers of lung and bronchus, stomach, oral cavity and pharynx, hodgkin lymphoma, kidney, thyroid, nervous system, non-hodgkin lymphoma, leukemias, and bladder.

**Additional file 2: Table S1**. Suicide Rate in Persons With Cancer by Age at Diagnosis.

**Additional file 3: Table S2**. Incidence of Suicide Among Cancer Patients ≥ 40 years old by Demographic and Tumor Characteristics.

**Additional file 4: Table S3**. Suicide Rates by Anatomic Site of Cancer in patients ≥ 40 years.

## Data Availability

The datasets generated and/or analysed during the current study are freely available in the SEER repository, [https://seer.cancer.gov/seerstat/].

## Abbreviations

AYA: Adolescent and young adult
SEER: Surveillance, Epidemiology, and End Results
SMR: Standardized mortality ratio
CI: Confidence interval
