# Supplementary figures and images for: Incidence of suicide among adolescent and young adult cancer patients: a population-based study
Source: Cancer Cell Int. 2021 Oct 18;21:540. doi: 10.1186/s12935-021-02225-y (PMC8522157; doi:10.1186/s12935-021-02225-y)

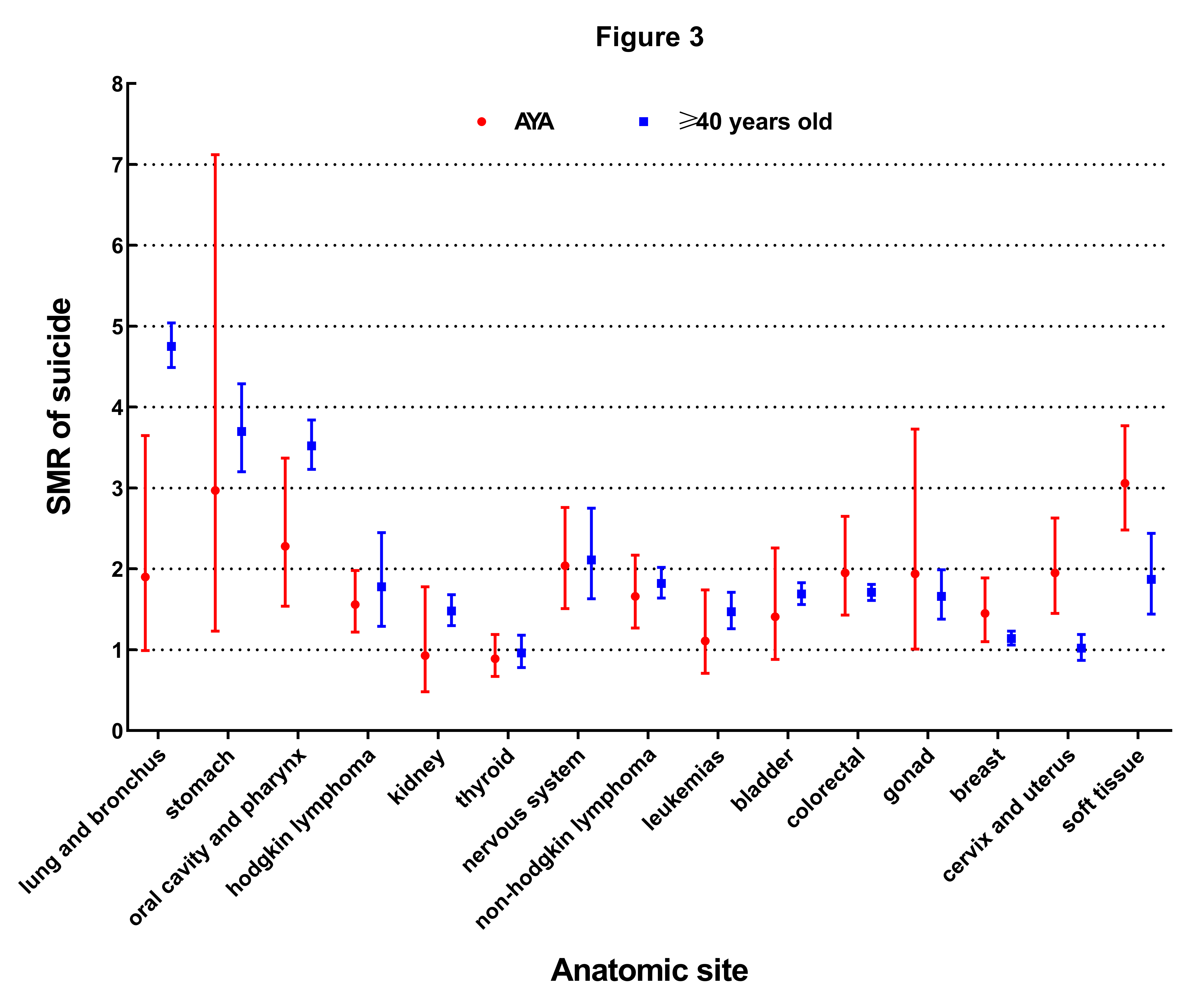

Supplement: Supplementary file 1 — Additional file 1: Figure 1. Comparison of suicide risk between AYA and older patients by cancer site. The distribution of suicide risk by tumor type was different between AYA patients and older patients. For AYA patients, SMRs of suicide were higher in those with cancers of colorectal, gonad, breast, cervix and uterus, and soft tissue. For older patients, higher SMRs of suicide were observed in those with cancers of lung and bronchus, stomach, oral cavity and pharynx, hodgkin lymphoma, kidney, thyroid, nervous system, non-hodgkin lymphoma, leukemias, and bladder. [file 12935_2021_2225_MOESM1_ESM.tif]
