# Supplementary material for: Incidence of suicide among adolescent and young adult cancer patients: a population-based study
Source: Cancer Cell Int. 2021 Oct 18;21:540. doi: 10.1186/s12935-021-02225-y (PMC8522157; doi:10.1186/s12935-021-02225-y)
Supplement: Supplementary file 2 — Additional file 2: Table S1. Suicide Rate in Persons With Cancer by Age at Diagnosis. [file 12935_2021_2225_MOESM2_ESM.docx]

| age^a^ | suicide rate in US | suicide rate in AYA | survival time | No of | SMR^c^ | CI | suicide rate in | suicide rate in |
| --- | --- | --- | --- | --- | --- | --- | --- | --- |
|  | general population^bc^ | cancer patients^b^ | (person-years) | Suicide |  |  | male patients^b^ | female patients^b^ |
| 15-19 | 8.44 | 13.45 | 356816.3 | 48 | 1.594 | 1.201-2.115 | 23.46 | 4.29 |
| 20-14 | 14.24 | 19.17 | 610317.6 | 117 | 1.346 | 1.123-1.631 | 28.71 | 11.69 |
| 25-29 | 14.6 | 16.89 | 994486.7 | 168 | 1.157 | 0.994-1.346 | 31.33 | 7.48 |
| 30-34 | 14.51 | 17.78 | 1467565.6 | 261 | 1.226 | 1.086-1.384 | 34.1 | 9.09 |
| 35-39 | 15.11 | 18.27 | 2118323.3 | 387 | 1.209 | 1.095-1.336 | 37.83 | 9.71 |

Table S1. Suicide Rate in Persons With Cancer by Age at Diagnosis

Abbreviations: SEER, Surveillance, Epidemiology, and End Results; SMR, standardized mortality ratio.

^a^ Analysis was limited to age groups for which at least 10,000 person years were accrued.

^b^ Per 100,000 person-years.

^c^ Reference population: general US population, 1969 to 2015.
