# Supplementary material for: Incidence of suicide among adolescent and young adult cancer patients: a population-based study
Source: Cancer Cell Int. 2021 Oct 18;21:540. doi: 10.1186/s12935-021-02225-y (PMC8522157; doi:10.1186/s12935-021-02225-y)
Supplement: Supplementary file 3 — Additional file 3: Table S2. Incidence of Suicide Among Cancer Patients ≥ 40 years old by Demographic and Tumor Characteristics. [file 12935_2021_2225_MOESM3_ESM.docx]

| characteristic | Patients with  cancer in SEER | | No.of Suicide | | Suicide per 100000  person years^a^ | SMR^ab^ | 95％CI |
| --- | --- | --- | --- | --- | --- | --- | --- |
|  | No. | % | No. | % |  |  |  |
| Sex |  |  | No |  |  |  |  |
| Male | 4,652,807 | 50.16 | 11439 | 82.73% | 48.2 | 1.66 | 1.63-1.69 |
| Female | 4,622,936 | 49.84 | 2388 | 17.27% | 8.7 | 1.20 | 1.15-1.25 |
| Race |  |  |  |  |  |  |  |
| White | 7,733,523 | 83.37 | 12815 | 92.68% | 29.6 | 1.61 | 1.59-1.64 |
| Black | 888,877 | 9.58 | 437 | 3.16% | 11.0 | 1.78 | 1.62-1.96 |
| Other | 653,343 | 7.04 | 575 | 4.16% | 17.4 | 1.95 | 1.80-2.12 |
| Marital status |  |  |  |  |  |  |  |
| Married | 5,196,577 | 56.02 | 7811 | 56.49% | 25.8 | 1.47 | 1.43-1.50 |
| Unmarried | 3,376,008 | 36.4 | 4880 | 35.29% | 32.3 | 1.89 | 1.84-1.95 |
| Unknown | 703,158 | 7.58 | 1136 | 8.22% | 29.7 | 1.78 | 1.68-1.89 |
| Stage at presentation |  |  |  |  |  |  |  |
| In situ | 624,439 | 6.73 | 735 | 5.32% | 15.7 | 0.88 | 0.82-0.94 |
| Localized | 3,639,232 | 39.23 | 5669 | 41.00% | 20.9 | 1.24 | 1.21-1.27 |
| Regional | 1,584,978 | 17.09 | 2336 | 16.89% | 29.8 | 1.71 | 1.64-1.78 |
| Distant | 1,674,265 | 18.05 | 1734 | 12.54% | 56.3 | 3.38 | 3.23-3.54 |
| Unstaged/Unknown | 1,752,829 | 18.9 | 3353 | 24.25% | 43.7 | 2.68 | 2.59-2.77 |
| No of primary |  |  |  |  |  |  |  |
| Single | 6,544,559 | 70.56 | 9961 | 72.04% | 29.9 | 1.74 | 1.71-1.78 |
| Multiple | 2,731,184 | 29.44 | 3866 | 27.96% | 22.2 | 1.36 | 1.32-1.41 |
| Year of diagnosis |  |  |  |  |  |  |  |
| 1973-1983 | 653,850 | 7.05 | 1626 | 11.76% | 35.4 | 1.95 | 1.85-2.04 |
| 1984-1994 | 1,263,160 | 13.62 | 3257 | 23.56% | 32.0 | 1.89 | 1.83-1.96 |
| 1995-2005 | 3,015,059 | 32.5 | 5023 | 36.33% | 23.4 | 1.38 | 1.34-1.42 |
| 2006-2015 | 4,343,674 | 46.83 | 3921 | 28.36% | 27.7 | 1.67 | 1.62-1.72 |
| All patients with cancer | 9,275,743 | 100 | 13827 | 100.00% | 27.4 | 1.62 | 1.59-1.65 |

Table S2. Incidence of Suicide Among Cancer Patients ≥ 40 years old by Demographic and Tumor Characteristics

Abbreviations: SEER, Surveillance, Epidemiology, and End Results; SMR, standardized mortality ratio.

^a^ Adjusted to the age distribution in the population served by the SEER program.

^b^ For the categories of sex and race, SMR reference population was the specific category in the US subpopulation ≥ 40 years (eg, the SMR for males is the observed number of suicides in male patients ≥ 40 years divided by the expected number of suicides based on the rate in men in the general population ≥ 40 years). For marital status, stage at presentation, number of primary tumors, and year of diagnosis, SMR reference population is the entire general US population≥ 40 years from 1969 through 2015.
