# Supplementary material for: Incidence of suicide among adolescent and young adult cancer patients: a population-based study
Source: Cancer Cell Int. 2021 Oct 18;21:540. doi: 10.1186/s12935-021-02225-y (PMC8522157; doi:10.1186/s12935-021-02225-y)
Supplement: Supplementary file 4 — Additional file 4: Table S3. Suicide Rates by Anatomic Site of Cancer in patients ≥ 40 years. [file 12935_2021_2225_MOESM4_ESM.docx]

| site^a^ | All ≥ 40 years cancer patients | | | | | ≥ 40 years male patients^e^ | | | ≥ 40 years female patients^e^ | | |
| --- | --- | --- | --- | --- | --- | --- | --- | --- | --- | --- | --- |
|  | No.of suicides | No.of patients | Suicide rate^bc^ | SMR^cd^ | 95% CI^cd^ | Suicide rate^bc^ | SMR^cd^ | 95% CI^cd^ | Suicide rate^bc^ | SMR^cd^ | 95% CI^cd^ |
| Bones and Joints | 14 | 6,569 | 41.5 | 2.26 | 1.34-3.81 | 74.4 | 2.51 | 1.42-4.41 | 12.9 | 1.41 | 0.35-5.64 |
| Brain and Other Nervous System | 56 | 78,297 | 48.5 | 2.11 | 1.63-2.75 | 93.1 | 2.38 | 1.81-3.14 | 9.7 | 1.09 | 0.49-2.43 |
| Breast | 699 | 1,047,464 | 8.4 | — | — | 0.0 | 0.00 | 0.00 | 8.4 | 1.14 | 1.06-1.23 |
| Cervix and Uterus | 159 | 257,614 | 7.7 | — | — | 0.0 | 0.00 | 0.00 | 7.7 | 1.02 | 0.87-1.19 |
| Colon and Rectum | 1114 | 705,433 | 28.7 | 1.71 | 1.61-1.81 | 52.1 | 1.81 | 1.70-1.93 | 8.4 | 1.28 | 1.09-1.49 |
| Gonad | 113 | 118,885 | 41.2 | 1.66 | 1.38-1.99 | 70.3 | 1.21 | 0.89-1.66 | 15.9 | 2.05 | 1.63-2.57 |
| Hodgkin Lymphoma | 37 | 18,937 | 32.3 | 1.78 | 1.29-2.45 | 64.7 | 2.05 | 1.47-2.86 | 4.1 | 0.53 | 0.13-2.12 |
| Kidney and Renal Pelvis | 232 | 166,888 | 26.3 | 1.48 | 1.30-1.68 | 44.5 | 1.49 | 1.30-1.72 | 10.5 | 1.40 | 0.99-1.98 |
| Larynx | 213 | 51,466 | 52.6 | 3.05 | 2.66-3.48 | 88.8 | 3.05 | 2.66-3.50 | 21.1 | 2.98 | 1.69-5.25 |
| Leukemia | 163 | 154,426 | 25.3 | 1.47 | 1.26-1.71 | 41.6 | 1.45 | 1.23-1.71 | 11.2 | 1.57 | 1.07-2.28 |
| Lung and Bronchus | 1148 | 875,402 | 78.8 | 4.75 | 4.49-5.04 | 145.4 | 5.19 | 4.88-5.52 | 20.7 | 2.86 | 2.41-3.40 |
| Non-Hodgkin Lymphoma | 362 | 235,787 | 31.0 | 1.82 | 1.64-2.02 | 54.3 | 1.91 | 1.70-2.14 | 10.6 | 1.47 | 1.14-1.89 |
| Nose, Nasal Cavity and Middle Ear | 16 | 9,096 | 35.6 | 2.03 | 1.24-3.31 | 63.3 | 2.12 | 1.26-3.58 | 11.5 | 1.56 | 0.39-6.24 |
| Oral Cavity and Pharynx | 521 | 146,272 | 60.6 | 3.52 | 3.23-3.84 | 109.2 | 3.64 | 3.33-3.99 | 18.4 | 2.52 | 1.85-3.42 |
| Skin excluding Basal and Squamous | 571 | 339,029 | 21.5 | 1.26 | 1.16-1.37 | 37.7 | 1.32 | 1.21-1.44 | 7.4 | 1.02 | 0.83-1.26 |
| Soft Tissue including Heart | 55 | 31,304 | 35.5 | 1.87 | 1.44-2.44 | 68.4 | 2.10 | 1.59-2.76 | 6.9 | 0.91 | 0.38-2.18 |
| Stomach | 180 | 123,688 | 60.6 | 3.70 | 3.20-4.29 | 112.3 | 3.92 | 3.36-4.57 | 15.5 | 2.48 | 1.56-3.93 |
| Thyroid | 89 | 103,721 | 27.3 | 0.96 | 0.78-1.18 | 50.7 | 1.15 | 0.88-1.51 | 6.8 | 0.76 | 0.54-1.05 |
| Urinary Bladder | 591 | 232,465 | 27.2 | 1.69 | 1.56-1.83 | 49.8 | 1.73 | 1.59-1.88 | 7.5 | 1.20 | 0.85-1.71 |
| All patients ≥ 40 years with single tumor | 9961 | 6,544,559 | 29.5 | 1.71 | 1.68-1.75 | 52.4 | 1.83 | 1.80-1.87 | 9.5 | 1.29 | 1.23-1.36 |
| General US population |  |  | 17.2 | 1.00 |  | 28.5 | 1.00 |  | 7.4 | 1.00 |  |

Table S3. Suicide Rates by Anatomic Site of Cancer in patients ≥ 40 years

Abbreviations: SMR, standardized mortality ratio.

^a^ Analysis was limited to tumor sites for which at least 100,000 person years were accrued.

^b^ Per 100,000 person-years.

^c^ Adjusted to the age, race, and sex distributions of patients with a single primary tumor.

^d^ Reference population: general US ≥ 40 years population, 1969 to 2015.

^e^ Sex-specific analysis, adjusted for age and race distributions of patients with single primary tumor.
